# Using the Extended Parallel Process Model to Examine the Nature and Impact of Breast Cancer Prevention Information on Mobile-Based Social Media: Content Analysis

**DOI:** 10.2196/13987

**Published:** 2019-06-24

**Authors:** Liang Chen, Xiaodong Yang, Lunrui Fu, Xiaoming Liu, Congyi Yuan

**Affiliations:** 1 Key Laboratory for Big Data Analysis and Simulation of Public Opinion School of Communication and Design Sun Yat-sen University Guangzhou China; 2 School of Journalism and Communication Shandong University Jinan China

**Keywords:** breast cancer, prevention information, mobile social media, EPPM

## Abstract

**Background:**

With the rise of mobile technology, an increasing number of people use mobile-based social media to access health information. Many scholars have explored the nature of health information on social media; however, the impact of such information on people was understudied.

**Objective:**

This study aimed to examine the nature and impact of health information on mobile-based social media. Specifically, we investigated how the levels of threat and efficacy of breast cancer prevention information affect individuals’ engagement with the information, such as readings and likes.

**Methods:**

Breast cancer prevention articles posted on a Chinese mobile-based social media platform (ie, WeChat Subscription Account [WeChat SA]) from January 1 to December 31, 2017, were extracted using the Python Web Crawler. We used content analysis and analysis of covariance to analyze our data.

**Results:**

The results revealed that the vast majority of titles and main bodies of the articles involved one of the extended parallel process model components: threat or efficacy.

**Conclusions:**

Breast cancer prevention information on WeChat SA was well designed. Both threat and efficacy significantly affected the number of readings, whereas only efficacy had a significant effect on the number of likes. Moreover, breast cancer prevention information that contained both high levels of threat and efficacy gained the largest number of readings and likes.

## Introduction

### Background

With the rise of mobile technology, an increasing number of people use mobile-based social media to access health information, for example, seeking social support from others and inquiring of medical professionals. In China, WeChat has become the most widely and frequently used mobile-based social media platform and is a crucial part of Chinese people’s daily lives [1]. In 2012, WeChat provided a platform called *Subscription Account* (SA). Similar to Facebook pages, people automatically receive information that they are interested in by following a certain SA. With the high incidence and mortality rate of breast cancer in China [2], many breast cancer–related SAs were created on WeChat to provide a variety of information regarding breast cancer prevention.

A number of studies have explored the nature of health information on social media. For example, Jiang and Beaudoin examined the content of antismoking information on social media based on 3 concepts: subjective norms, perceived risk, and self-efficacy [3]. They found that most content is related to perceived risk, followed by the other 2 factors. Zhang and He examined 1352 messages posted in a Facebook diabetes group and found that health information mainly comprises personal experience, opinions, and advice [4]. Shi and Chen examined the content of all 7215 messages posted in an HIV/AIDS Weibo group and found that the health information in People living with HIV/AIDS Weibo group mainly included emotional, informational, and instrumental support [5]. Although many scholars have explored the nature of health information on social media, the impact of such information on people was understudied.

Therefore, this study intended to examine both the nature and impact of breast cancer–related information on WeChat SA based on the extended parallel process model (EPPM) [6,7]. Some scholars have suggested that media content may not directly affect people’s attitudes or behaviors but may instead stimulate them to pay more attention to, and engage more in, related information first [8]. Thus, instead of exploring the direct effect of social media on individuals’ breast cancer preventive behavior, this study sought to investigate how the level of threat and efficacy of breast cancer prevention information affects individuals’ engagement with the information.

### The Extended Parallel Process Model

The fundamental aim of health communication is to deliver knowledge to people to assist them in response to potential diseases or health problems. In modern society, the media is acknowledged as the most prevalent provider of health information, and it is effective in raising public awareness of diseases and promoting precautionary action [9]. Considering the potential impact of the media on public reactions to health issues, this study endeavors to investigate how health messages are constructed in the media and how health information influences people’s engagement with information. Thus, this study adopts the EPPM as a theoretical basis.

The EPPM is a predominant message design theory for investigating individuals’ reactions to fear appeals [6]. According to the EPPM, to persuade specific groups of people, threat messages should be first provided to attract individuals’ attention and stimulate their perception of threat. More specifically, perceived threat includes 2 elements: perceived severity and susceptibility [10]. Perceived severity refers to the perception of seriousness or magnitude of a threat, whereas perceived susceptibility refers to the likelihood of suffering from the threat based on one’s belief. After individuals evaluate the extent to which they are likely to experience the threat and the seriousness of the threat, they should receive feasible recommendations that could increase their perceived efficacy. Perceived efficacy also comprises 2 dimensions: perceived self-efficacy and response efficacy. To be specific, perceived self-efficacy refers to an individual’s beliefs regarding his or her ability to perform recommended behaviors, whereas perceived response efficacy refers to personal beliefs regarding the effectiveness of the recommended behaviors to avoid hazards. Ideally, when the audience has a higher level of perceived efficacy than that of threat, they will enter the danger control process in which they perform protective behaviors to avert hazards.

A number of empirical studies have documented that fear appeal messages are effective in motivating individuals to perform certain health behaviors. For example, Chen and Yang (2018) found that messages containing a high level of threat and efficacy increased women’s intentions to adopt recommended practices, such as breast self-examination [11]. Kotowski et al found that well-designed fear appeal messages can motivate people to use hearing protection aids [12]. Some studies have indicated that people who are exposed to these messages only once may not change their behaviors directly. However, the effectiveness of fear appeal messages might be realized by multiple interactions with the information instead of one exposure to the information [13]. Several studies have pointed out that fear appeal messages may not directly lead people to protective behavior but may motivate them to seek and share relevant information and engage in discussion about a particular issue first [8,14,15]. Thus, we applied the EPPM to examine how breast cancer–related information can affect people’s engagement with information.

### Engagement With Social Media Content

Social media content usually provides more information than does traditional media. The message itself, the social media content, usually includes some social cues that refer to system- and user-generated cues, such as the number of readings and the number of likes on WeChat SA [16]. Some scholars have indicated that social cues, including the number of readings and likes, have become common indicators of social media campaigns’ reach and awareness [17,18]. A higher number of readings and likes indicate a high level of media effects. Thus, social cues have been used as proxies to represent the effectiveness of mobile-based social media campaigns [16]. On the basis of the above, this study intended to examine how the threat and efficacy components in breast cancer prevention messages affect the number of readings and likes. We proposed the following research questions (RQs):

- *RQ1*: How do the titles of articles on breast cancer–related WeChat SAs present (1) threat and (2) efficacy?
- *RQ2*: How do the main bodies of articles on breast cancer–related WeChat SAs present (1) threat and (2) efficacy?
- *RQ3*: How does the threat of article titles affect the number of readings?
- *RQ4*: How does the efficacy of article titles affect the number of readings?
- *RQ5*: How does the threat of the main bodies of the article affect the number of likes?
- *RQ6*: How does the efficacy of the main bodies of the article affect the number of likes?

## Methods

### Data Extraction

All articles regarding breast cancer prevention posted in breast cancer–related WeChat SAs from January 1 to December 31, 2017, were extracted using the Python Web Crawler in September 2018. The unit of analysis for this study was a single article. Commercial advertisements and articles not related to breast cancer prevention were excluded from the sample. Hence, the final sample size was 537 articles. The content, length of the title and main body, number of readings and likes, and video were retrieved.

### Content Analysis

Manual content analysis was used in the current research. Specifically, 3 trained coders, who are native Chinese speakers, were recruited to conduct the coding procedure. First, the 537 article titles were coded as 0 (absent) and 1 (present) in the 2 variables: threat and efficacy. Krippendorff alpha tests revealed an acceptable level of intercoder reliability for the 2 variables: .87 for threat and .91 for efficacy. The main bodies of the article were coded as 0 (low), 1 (medium), and 2 (high) for the 2 variables: threat and efficacy. The values of Krippendorff alpha were .81 for threat and .83 for efficacy, which is also acceptable.

## Results

### The Nature of Breast Cancer Prevention Information on WeChat Subscription Accounts

First, the results showed that 82.1% (441/537) article titles involved one of the EPPM components: threat or efficacy. To be specific, 63.9% (343/537) article titles contained efficacy and 26.4 % (142/537) article titles contained threat. In terms of the main bodies of the articles, 75.2% (404/537) articles provided medium and high levels of threat, whereas only 49.7% (267/537) articles contained medium and high levels of threat.

### The Impact of Breast Cancer Prevention Information on WeChat Subscription Accounts

**Figure 1:**
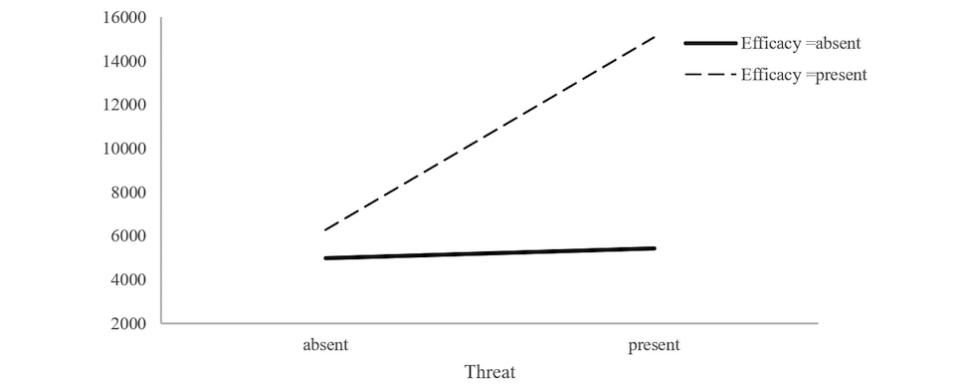
Interaction effect between threat and efficacy upon the number of readings.

In addition, a series of analysis of covariance (ANCOVAs) was conducted to address *RQ3* to *RQ6*. First, an ANCOVA was performed to examine how the threat and efficacy of article titles affect the number of readings, with title length serving as a covariate. The results revealed a significant main effect for threat, where titles with the threat component had a greater number of readings (mean 8448.73, SD 8286.11) and those without the threat component had a lower number of readings (mean 5957.64, SD 4852.52), *F*_1,532_=62.99, *P*<.001, partial *η*^2^=0.11 (see Tables 1 and 2). The results also showed a significant main effect for efficacy, which indicated that titles with the efficacy component tended to have a greater number of readings (mean 7477.39, SD 6346.04) than did those without the efficacy component (mean 5094.04, SD 5155.45), *F*_1,532_=85.87, *P*<.001, partial *η*^2^=0.14 (see Tables 1 and 2). Furthermore, the results revealed a significant interaction effect between threat and efficacy, *F*_1,532_=51.51, *P*<.001, partial *η*^2^=0.09. Figure 1 presents a plot of the obtained mean scores. An examination of this interaction effect indicates that the number of readings was highest when both threat and efficacy were presented in the article titles.

**Table 1:** Descriptive statistics for the number of readings (N=537).

Presence of threat and efficacy		Mean (SD)	n (%)
**Threat absent**			
	Efficacy absent	4881.70 (3969.35)	96 (17.9)
	Efficacy present	6303.09 (5061.33)	299 (55.7)
	Total	5957.64 (4852.52)	395 (73.6)
**Threat present**			
	Efficacy absent	5302.03 (6112.63)	98 (18.3)
	Efficacy present	15457.27 (8247.67)	44 (8.2)
	Total	8448.72 (8286.11)	142 (26.4)
**Total**			
	Efficacy absent	5094.03 (5155.45)	194 (36.1)
	Efficacy present	7477.39 (6346.04)	343 (63.9)
	Total	6616.36 (6048.11)	537 (100)

**Table 2:** Threat×efficacy factorial analysis of variance for the amount of reading.

Variable	*F* test (df)	*P* value	Partial *η*^2^
Title length	11.03 (1)	.001	0.02
Threat	62.99 (1)	<.001	0.11
Efficacy	85.87 (1)	<.001	0.14
Threat×Efficacy	51.51 (1)	<.001	0.09

Additionally, another ANCOVA was performed to explore how the level of threat and efficacy presented in the main bodies of the articles affected the number of likes, with videos and article length serving as covariates. The results revealed that the main effect for threat was not significant (*F*_2,526_=.65; *P*=.52; partial *η*^2^=0.002). However, the main effect for efficacy was significant (*F*_2,526_=5.45; *P*=.005; partial *η*^2^=0.02), where articles with a high level of efficacy (mean 46.75, SD 63.18) had a greater number of likes than did those with medium (mean 34.19, SD 47.36) and low levels of efficacy (mean 26.68, SD 32.82; see Tables 3 and 4).

**Table 3:** Descriptive statistics for the number of likes (N=537).

Level of threat and efficacy		Mean (SD)	n (%)
**Low threat**			
	Low efficacy	25.59 (27.71)	87 (16.2)
	Medium efficacy	29.68 (31.61)	60 (11.2)
	High efficacy	37.69 (38.18)	123 (22.9)
	Total	32.01 (33.97)	270 (50.3)
**Medium threat**			
	Low efficacy	35.63 (43.89)	30 (5.6)
	Medium efficacy	39.90 (40.21)	48 (8.9)
	High efficacy	41.84 (43.68)	81 (15.1)
	Total	40.08 (42.49)	159 (29.6)
**High threat**			
	Low efficacy	15.88 (32.51)	16 (3.0)
	Medium efficacy	34.04 (83.64)	23 (4.3)
	High efficacy	68.68 (102.19)	69 (12.9)
	Total	53.48 (93.08)	108 (20.1)
**Total**			
	Low	26.68 (32.82)	133 (24.8)
	Medium	34.19 (47.36)	131 (24.4)
	High	46.75 (63.18)	273 (50.8)
	Total	38.72 (53.93)	537 (100)

**Table 4:** Threat×efficacy factorial analysis of variance for intention to the number of likes.

Variable	*F* test (df)	*P* value	Partial *η*^2^
Article length	10.10 (1)	.002	0.02
Video	0.97 (1)	.32	0.00
Threat	0.65 (2)	.52	0.00
Efficacy	5.45 (2)	.005	0.02
Threat×Efficacy	2.53 (4)	.04	0.02

**Figure 2:**
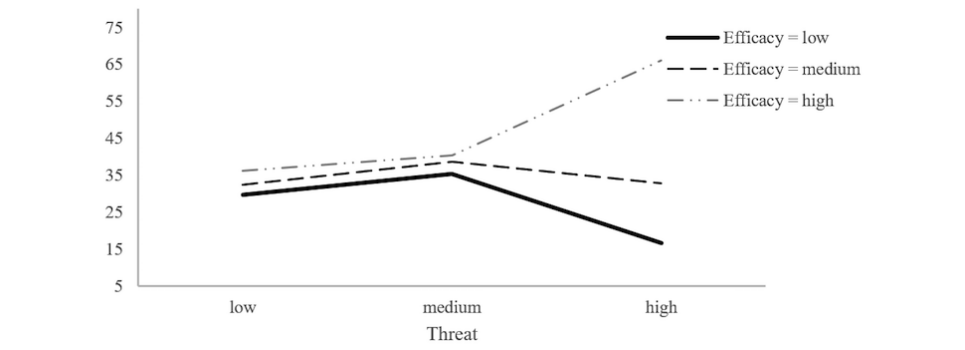
Interaction effect between threat and efficacy upon the number of likes.

The results revealed that the threat×efficacy interaction effect (*F*_4,526_=2.53; *P*=.04; partial *η*^2^=0.02) was significant. Figure 2 presents a plot of the obtained mean scores, which indicated that for the main bodies of the articles with low or medium levels of threat, regardless of the level of efficacy, the number of likes remained stable and relatively low. Similarly, for the main bodies of the articles with a high level of threat, coupled with low or medium levels of efficacy messages, the number of likes was small. However, for the main bodies of the articles with high levels of threat and efficacy, the number of likes was highest.

## Discussion

### Principal Findings

This study examined the nature and impact of mobile-based social media messages. Single exposure to information may not lead to immediate behavior change. Media content may stimulate people’s information attention and engagement (ie, liking, sharing, and commenting) [8,14,15,19], which promotes actual behaviors [20]. As such, this study explored how mobile-based social media messages affect individuals’ engagement with the information based on the EPPM.

First, the WeChat SA article titles with a threat or efficacy component had a greater number of readings than did those without threat and efficacy components. Moreover, the number of readings was highest when both threat and efficacy were present in the article titles. This is consistent with the EPPM model. Titles containing the threat of breast cancer will attract people’s attention and arouse their feelings of fear [6,21], which will motivate them to seek preventive solutions. In this regard, once the titles contain the efficacy component, people will perceive that the article will provide effective measures. Thus, they are more likely to read these articles.

In addition, the results revealed that the main bodies of the articles containing a high level of efficacy received a greater number of likes than did those with medium and low levels of efficacy, whereas the level of threat did not significantly affect the number of likes. It is plausible that most people who followed the WeChat SA related to breast cancer were aware of the risk of breast cancer before reading the articles. Moreover, the article titles may have aroused their feelings of fear. In this regard, they prefer to find effective and feasible measures to reduce their fear and anxiety instead of increasing perceived risk about diseases.

More interestingly, the main bodies of the articles that contain high levels of threat and efficacy gained the largest number of likes. However, those with low levels of efficacy but high levels of threat did not lead to public agreement. This is in line with the EPPM. People who experience an overly high perceived threat believe that they are incapable of adopting protective behaviors or their beliefs fall under the ineffectiveness of such behaviors. Thus, they would subsequently enter the fear control process and engage in defensive avoidance or reaction behaviors [7]. In contrast, when people have a higher perception of efficacy than threat, they enter the danger control process and change their perceptions, attitudes, intentions, and even actual behaviors to cope with the threat according to the recommended responses [10].

### Implications and Limitations

This study is an initial attempt to examine the nature and impact of mobile-based social media messages on people’s engagement with information. First, breast cancer prevention information on the most popular Chinese mobile-based social media platform (ie, WeChat SA) was analyzed and found to be well designed. However, instead of examining the direct effect of social media on individuals’ actual behavior, this study investigated how threat and efficacy of breast cancer prevention articles on WeChat SAs affect the number of readings and likes. This provides a new approach or perspective to examine the effects of social media–based health campaigns,

Despite its significant contributions, this study is not without limitations. First, only breast cancer prevention–related WeChat SA articles were examined in this study, which may limit generalization of the findings to other diseases and health conditions. Future studies should examine mobile-based social media content regarding other diseases. Second, only the number of readings and likes were included as indicators of individuals’ engagement with the information in this study. Other social cues, such as the number of comments and retweets, may also represent the level of engagement with the information. Thus, the content of comments and the number of retweets could be considered. Finally, this study examined the effects of mobile-based social media only on individuals’ engagement with the information rather than their actual behaviors. Although previous studies have indicated the link between engagement with information and actual behavior [8], future research should examine whether and how engagement with information affects actual preventive behavior.

## Abbreviations

ANCOVA: analysis of covariance
EPPM: Extended Parallel Process Model
RQ: research question
SA: Subscription Account
